# THE MANY VOICES OF CARE PARTNERS ACROSS THE CONTINUUM OF CARE

**DOI:** 10.1093/geroni/igac059.1124

**Published:** 2022-12-20

**Authors:** Aimee Fox, Abigail Latimer, Deborah Waldrop

## Abstract

Caregiving and care-sharing is a dynamic, stressful, and physically demanding responsibility, often leading to adverse psychological and physical outcomes. Caregiving for chronic illness and disease involves increasing complexity and scope of responsibilities and expectations. Utilizing strengths-based approaches and a variety of qualitative methods, this symposium highlights the many voices of care partners across the continuum of care; pre-, during and post-caregiving. First, Latimer and colleagues will present a case study of an older adult with multiple chronic illnesses, offering insight into anticipating care needs and coping with daily stressors of multi-morbidity in late life. Second, Morgan and colleagues will present findings from interviews with care partners on supporting the inner strength of those recently diagnosed with mild cognitive impairment. Third, Fox and colleagues share results from a dyadic, multi-modal intervention for pain management. Care partners who both experience persistent pain discuss changes in their relationship as a result of participating in the intervention together. Fourth, Wladkowski discusses caregiver’s perspectives on live discharge and re-enrollment into hospice care. Her findings demonstrate how the anticipated “end” isn’t always the end for ADRD caregivers. Fifth, Buck and colleagues will share caregivers’ responses to a psychotherapy intervention for the treatment of complicated grief post-death of the care partner. Discussant Deborah Waldrop will contextualize these findings and offer suggestions for future research and interventions to enhance care partners’ well-being across the continuum of chronic illness and care.

